# Gut colonization with multidrug resistant organisms in the intensive care unit: a systematic review and meta-analysis

**DOI:** 10.1186/s13054-024-04999-9

**Published:** 2024-06-28

**Authors:** Madison R. Heath, Weijia Fan, Cheng-Shiun Leu, Angela Gomez-Simmonds, Thomas Lodise, Daniel E. Freedberg

**Affiliations:** 1 Division of Digestive and Liver Diseases, Columbia University Medical Center, New York, NY USA; 2 Mailman School of Public Health, Columbia University, New York, NY USA; 3 Albany College of Pharmacy and Health Sciences, Albany, NY USA

**Keywords:** ICU, MDRO, Drug resistance, Intestinal colonization, Microbiome

## Abstract

**Background:**

Gut colonization with multidrug-resistant organisms (MDRO) frequently precedes infection among patients in the intensive care unit (ICU), although the dynamics of colonization are not completely understood. We performed a systematic review and meta-analysis of ICU studies which described the cumulative incidence and rates of MDRO gut acquisition.

**Methods:**

We systematically searched PubMed, Embase, and Web of Science for studies published from 2010 to 2023 reporting on gut acquisition of MDRO in the ICU. MDRO were defined as multidrug resistant non-*Pseudomonas* Gram-negative bacteria (NP-GN), *Pseudomonas* spp., and vancomycin-resistant *Enterococcus (*VRE). We included observational studies which obtained perianal or rectal swabs at ICU admission (within 48 h) and at one or more subsequent timepoints. Our primary outcome was the incidence rate of gut acquisition of MDRO, defined as any MDRO newly detected after ICU admission (i.e., not present at baseline) for all patient-time at risk. The study was registered with PROSPERO, CRD42023481569.

**Results:**

Of 482 studies initially identified, 14 studies with 37,305 patients met criteria for inclusion. The pooled incidence of gut acquisition of MDRO during ICU hospitalization was 5% (range: 1–43%) with a pooled incidence rate of 12.2 (95% CI 8.1–18.6) per 1000 patient-days. Median time to acquisition ranged from 4 to 26 days after ICU admission. Results were similar for NP-GN and *Pseudomonas* spp., with insufficient data to assess VRE. Among six studies which provided sufficient data to perform curve fitting, there was a quasi-linear increase in gut MDRO colonization of 1.41% per day which was stable through 30 days of ICU hospitalization (R^2^ = 0.50, *p* < 0.01).

**Conclusions:**

Acquisition of gut MDRO was common in the ICU and increases with days spent in ICU through 30 days of follow-up. These data may guide future interventions seeking to prevent gut acquisition of MDRO in the ICU.

**Supplementary Information:**

The online version contains supplementary material available at 10.1186/s13054-024-04999-9.

## Introduction

Gut colonization by multi-drug resistant organisms (MDRO) is associated with increased risk for organism-specific infections (e.g., VRE gut colonization and subsequent VRE infection) in the intensive care unit (ICU), and is associated with increased risk of death and all-cause infection [1–6]. Prior studies describe high rates of gut MDRO colonization in ICUs around the world, yet leave lack of precision in the estimates of incidence of colonization, and in its dynamics [7–11]. These studies suggest that rates of colonization may vary by organism, by geographic region, and by time period [9, 12]. Recently, several comprehensive meta-analyses have found high rates of systemic infection after gut MDRO colonization [3, 7, 12, 13]. However, no recent meta-analysis has examined the dynamic acquisition of gut MDRO as a function of time spent in the ICU (i.e., requiring studies which utilized longitudinal samples).

A comprehensive understanding of the dynamics of MDRO gut colonization among ICU patients is required to inform the design and endpoints of clinical trials that evaluate measures directed at preventing gut colonization and subsequent infections by MDROs. Some preventative measures are already being developed. For example, microbiome restitution therapies have been proposed as a novel approach for reducing rates of gut MDRO colonization [4, 14, 15] and can be effective in preventing recurrent *C. difficile* infection [16]. FMT has also been trialed to prevent recurrent systemic infections among those who are persistently gut MDRO colonized [14, 15]. If other gut microbiome-derived therapies are to be efficiently tested in the ICU, future ICU trials will need to know which patients to target, when to dose the therapies, and when to assess the results.

Given this gap in the literature, this meta-analysis quantified the incidence of acquisition of gut MDRO between ICU admission and discharge, generated a pooled incidence rate for acquisition of gut MDRO per 1000 patient-days, and described the dynamics of acquisition of gut MDRO colonization based on cohort studies which performed longitudinal samples at predetermined timepoints.

## Methods

### Search strategy and selection criteria

PubMed, Embase, and Web of Science were searched for relevant publications. The search strategy was adapted to fit each database’s research criteria and search terms were determined using the CoCoPop methodology [17] (Supplemental Table S1). The search dates were restricted to approximately the last 10 years (January 1, 2010 through November 8, 2023) based on a prior study showing substantive differences in rates of MDRO gut colonization comparing pre- versus post-2010 published studies [12]. Studies were required to report on MDRO gut colonization based on stool samples, perianal, or swabs that were gathered at ICU admission (within 48 h) and at one or more subsequent timepoint. Studies that did not report the prevalence of gut colonization on admission to the ICU, or which did not report a measure of the time between admission to gut acquisition of MDRO were excluded. To enhance generalizability, studies were further required to perform sampling on consecutive ICU patients (i.e., they were excluded if they focused on a specific subset of ICU patients, such as cirrhotic or immunocompromised patients), and to report on at least 50 participants at risk for gut MDRO colonization. Because we sought to report on the “natural history” of colonization in the ICU, all studies with an intervention were excluded, including studies which tested specific antibiotic regimens or selective decontamination of the digestive tract (SDD). Abstracts or studies lacking a published version of the full text in English were also excluded. The meta-analysis was registered with PROSPERO, CRD42023481569.

### Definition of MDRO

The definition for MDRO was based on World Health Organization (WHO) [18] guidance related to emerging infections; from the list of WHO organisms, we selected those that were known to be gut colonizers. These included non-*Pseudomonas* gram negative bacteria (NP-GN) with multidrug resistance (operationalized as resistance to third generation cephalosporins with or without carbapenem resistance and including Enterobacteriaceae such as *Klebsiella pneumoniae* or *Escherichia coli*), multidrug resistant *Pseudomonas* spp. (PA) with multidrug resistance (operationalized as resistance to two or more clinically significant antibiotic classes, as described by each included study), and vancomycin-resistant *Enterococcus* (VRE). For the purpose of this meta-analysis, these organisms were collectively classified as MDRO.

### Data Extraction and synthesis

Data extraction followed PRISMA recommendations for meta-analyses [19]. Studies were uploaded into Covidence for review [20]. After duplicates were removed, titles and abstracts were screened independently by two researchers (M.R.H., D.E.F.) to identify those that potentially met the inclusion criteria. The full texts of these potentially eligible studies were then retrieved and independently assessed for eligibility by the same two researchers. Disagreements were resolved by consensus. Pre-specified data was extracted and recorded in an Excel spreadsheet (M.R.H.) and independently reviewed for accuracy (D.E.F.).

### Primary outcome

The primary outcome was the incidence rate of gut acquisition of MDRO. It was defined as the number of newly detected MDRO cases over the total length of ICU stay beginning 48 h after ICU admission for all patient-time at risk. A patient was considered colonized if an MDRO was detected from a single swab, regardless of whether testing was repeated (and regardless of the results of repeat testing). Since several studies did not report incidence rate directly and had different follow up times for measuring the outcome, reported median or mean time to acquisition of MDRO and ICU length of stay were used to estimate person-time at risk as described in the Supplemental Methods. Patients who were colonized at ICU admission were not considered at risk for acquiring MDRO, so the at-risk population used for the acquired incidence was calculated by subtracting those with baseline colonization from the total population. Both the study-specific and pooled incidence rates for acquisition of MDROs per 1,000 patient-days were estimated. The prevalence of MDRO gut colonization upon admission to the ICU (within 48 h) and the incidence of newly acquired colonization during the ICU stay were also recorded. When reported, the prevalence of colonization in the ICU at specific timepoints was recorded for up to 30 days after ICU admission (e.g., proportion colonized on ICU Day 7, proportion colonized on ICU Day 14, etc.).

### Additional measures

For each study, we recorded the colonizing organism, sample type (perianal versus rectal), frequency of screening, organism identification methods, and antibiotic susceptibility testing methods. Additional study characteristics were recorded including inclusion criteria, geographic location, data collection period, study design, and type of ICU. Patient population characteristics including age and sex were recorded for each study.

### Statistical analysis

Measures of colonization were calculated including the prevalence of gut colonization on ICU admission, the incidence of patients who acquired gut MDRO during ICU hospitalization with their median or mean time to acquisition, and the incidence rate for acquisition of gut MDRO. The pooled incidence rates were estimated using a random-effects model using the inverse-variance weighting method. Confidence intervals for incidence rates were estimated using normal approximation. Stratified incidence rates were estimated based on pre-specified factors including organism type, sampling approach (weekly or more than weekly), country, data collection period, organism identification methods, and excluding the largest study. Study heterogeneity was quantified using the I^2^ statistic, which describes the proportion of variation across studies that can be explained by study heterogeneity rather than chance. Cochran’s Q test was conducted to assess the heterogeneity between studies. Publication bias was assessed using a funnel-plot and Egger’s test [21]. Finally, linear regression was performed on studies which reported prevalence of colonization at multiple timepoints to quantify the association between the outcome and days spent in the ICU. Analyses were conducted in R version 3.4.2.

### Quality and risk of *Bias*

Risk of bias and quality assessment was performed using the Quality Assessment Tool of the National Institutes of Health for Observational Cohort and Cross-Sectional studies [22]. The individual item and total quality rating for each study was recorded (Supplemental Methods). Study quality was depicted using a traffic light plot.

## Results

### Study selection

**Fig. 1:**
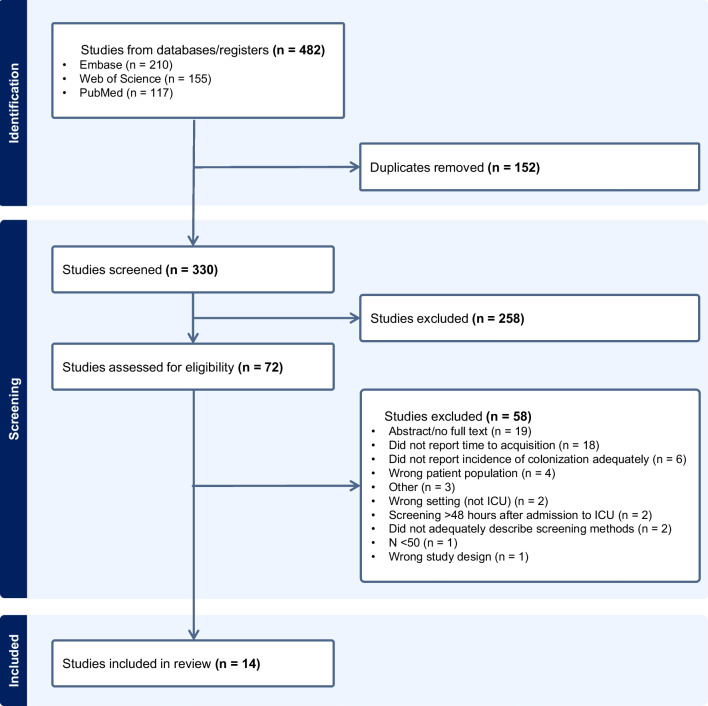
PRISMA flow diagram

A total of 482 studies were initially identified through the database search. Of these, 152 were removed as duplicates and 258 did not meet criteria based on abstract screening. Full text review was performed for 72 studies, out of which 58 were excluded, most often because they did not report MDRO colonization longitudinally (e.g., reported baseline colonization but did not report a measure of time to acquisition of colonization in the ICU) (Fig. 1).

### Study characteristics

The final meta-analysis comprised 14 studies consisting of a total of 37,305 patients (Table 1). Thirteen studies reported on NP-GN, three reported on *Pseudomonas*, and one reported on VRE. Most of the studies (11/14) were based in the E.U. or U.S. and most were conducted pre-2013 (10/14). Patient age ranged from 49 to 65.3 with a modest male sex predominance (median 61.5% male). Matrix assisted laser desorption/ionization (MALDI) was the most common method used for organism identification, with or without disk diffusion testing for antimicrobial susceptibility (Supplemental Table S2).

**Table 1:** Characteristics of included studies

Study	Location	Period	Type of ICU	Age (years)	Sex (male, %)
Ajao [32]	United States	2001–09	MICU; SICU	55.7 ± 15.6	55
Alves [33]	France	2011	MICU	64 (51–76)	56
Boutrot [34]	France	2015–17	SICU	63 (49–72)	72
Gomez-Zorrilla [35]	Spain	2012–13	MICU; SICU	65.3 ± 13.3^†^ & 62.2 ± 14.3^‡^	64
Grohs [23]	France	2011	MICU; SICU	64.2 ± 18.3	59
Jolivet [25]	France	1997–2015	MICU; SICU	59.4 (44.4–72.0)	64
Marchenay [36]	France	2011	MICU; SICU	65.3 ± 14.4^†^ & 7.4 ± 17.6^‡^	69
Papadimitriou-Olivgeris [37]	Greece	2010–11	ICU	56.4 ± 19.0	67
Poignant [38]	France	2008–10	MICU; SICU	63 (49–75)	63
Qin [39]	China	2018	Neuro	53.7 ± 17.8^†^ & 56.7 ± 16.6^‡^	70
Razazi [40]	France	2010–11	MICU	64 (49–76)^†^ & 64 (50–75)^‡^	60
Sharma [41]	India	2019–20	MICU; SICU	51 (26–62)^†^ & 50 (32–63)^‡^	58
Thiébaut [42]	France	2005–06	MICU; SICU	59 (47–72)	59
Torres-Gonzalez [43]	Mexico	2014	ICU	49 (32–64)	44

MICU = medical intensive care unit; SICU = surgical intensive care unit; Neuro = Neurological intensive care unit; Age was reported as “Mean ± SD” or “Median (interquartile range)” depending on what was reported in the study. Some studies only reported age based on colonization status: † indicates the subgroup that was colonized with a multidrug resistant organism and ‡ indicates the subgroup and that was not colonized

### Gut colonization with MDRO

**Fig. 2:**
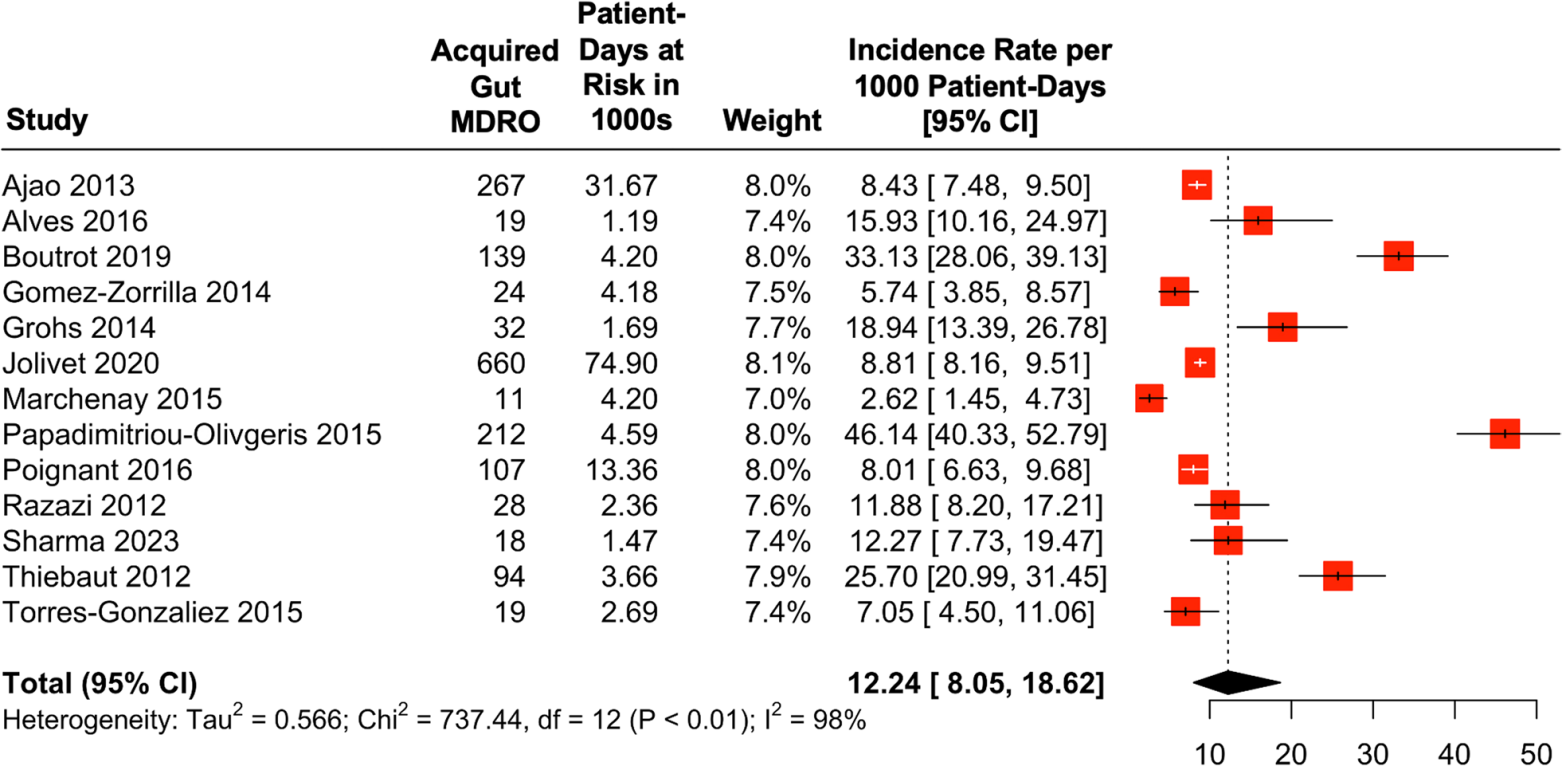
Forest plot of incidence rate of gut acquisition of MDRO per 1000 patient-days

The cumulative prevalence of gut colonization at the time of ICU admission (within 48 h) was 8% (median: 10%, IQR: 7–12%) (Table 2 and Supplemental Figure S1). The cumulative incidence of MDRO gut acquisition during ICU hospitalization (i.e., the proportion of patients who were negative for gut MDRO at admission and subsequently positive) was a mean of 5% (range 1–43%) and a median of 7% (IQR 6–15%). The median time to acquisition of MDRO ranged from 4 to 26 days. The overall incidence rate for acquisition of gut MDRO was 12.2 (95% CI 8.1–18.6) per 1,000 patient-days of ICU stay (Fig. 2). Significant heterogeneity across studies was observed (I^2^ = 98%, *p* < 0.01).

**Table 2:** Outcomes of interest for included studies

Study	Organism	N	Admission colonization N (%)	Acquired colonization N (%)	Median time to acquisition (days)	ICU length of stay (days)			Acquisition incidence rate per 1000 patient-days (95% CI)
						Non-colonized	Colonized	All	
Ajao [32]	NP-GN	8437	786 (9%)	267 (3%)	8 (4–15)	4 (2–8)	11 (5–24)	–	8.4 (7.4–9.5)
Alves [33]	NP-GN	309	25 (8%)	19 (7%)	7 (4–15)	4 (3–6)	12 (8–23)	–	15.9 (10.2–25.0)
Boutrot [34]	NP-GN	352	33 (9%)	87 (27%)	14 (6–20)	13 (8–24)	25 (16–46)	–	20.6 (16.7–25.4)
	PA		7 (2%)	52 (15%)	13 (7–21)				11.6 (8.8–15.2)
Gomez-Zorrilla [35]	PA	414	23 (6%)	24 (6%)	9 (7.5–12)	10.8 ± 9.2	15.6 ± 10.5	–	5.7 (3.9–8.6)
Grohs [23]	NP-GN	269	61 (23%)	32 (15%)	5.5 (4–8.3)	–	–	8.6^*^	18.9 (13.4–26.8)
Jolivet [25]	NP-GN	23,423	1667 (7%)	660 (3%)	8 (5–13)	–	–	3.3 (1.2–8.8)	8.8 (8.2–9.5)
Marchenay [36]	NP-GN	347	11 (3%)^†^	6 (2%)	15.3 ± 10.7	12.4 ± 12.5	–	–	1.4 (0.6–3.2)
	PA			5 (1%)					1.2 (0.5–2.9)
Papadimitriou-Olivgeris [37]	NP-GN	481	59 (12%)	181 (43%)	9.3 ± 5.5	–	12.7 ± 13.8	16.4 ± 20.3	32.1 (27.8–37.2)
	VRE		63 (13%)	31 (7%)	16.1 ± 8.9		16.6 ± 21.4		4.6 (3.2–6.4)
Poignant [38]	NP-GN	1,209	24 (2%)	107 (9%)	14 (7–21)	11 (7–17)	18 (10–30)	–	8.0 (6.6–9.7)
Qin [39]	NP-GN	243	37 (15%)^‡^	39 (19%)^‡^	7 (7–7)	–	–	–	–
Razazi [40]	NP-GN	531	82 (15%)	28 (6%)	9 (8–20)	5 (3–10)	9 (4–19)	–	11.9 (8.2–17.2)
Sharma [41]	NP-GN	192	19 (10%)	18 (10%)	4 (4–5.5)	9 (6–16)	12 (8–22)	–	12.3 (7.7–19.5)
Thiébaut [42]	NP-GN	768	74 (10%)	94 (14%)	7 (3–11)	–	–	5 (3–11)	25.7 (21.0–31.5)
Torres-Gonzalez [43]	NP-GN	330	36 (11%)	19 (6%)	26 (12–46)	8 (3–16)	15 (8–28)	–	7.1 (4.5–11.1)

NP-GN = non-*Pseudomonas* gram-negative organism. PA = *Pseudomonas aeruginosa*. VRE = vancomycin-resistant *Enterococcus*. ICU length of stay was reported as “Mean ± SD” or “Median (interquartile range)” depending on what was reported in the study and separated by colonization status depending on what was reported. A “–” indicates the value was not reported and/or incalculable. † indicates that the number of cases on admission was not separated by organism. ‡ indicates that the number of cases was not separated by rectal vs nasopharyngeal swab. *indicates a mean with no standard deviation reported. Admission colonization is a prevalence out of the total population while acquired colonization is an incidence out of the population at risk

### Stratified Analyses

To assess which factors may drive heterogeneity, the studies were stratified based on type of organism (NP-GN vs. *Pseudomonas*), approach to screening (sampling less vs. more than weekly), study location (Europe vs. elsewhere), data collection period (pre- vs. post-2013), and organism identification (MALDI vs. non-MALDI) (Table 3 & Supplemental Figure S2–S7). An additional analysis was performed excluding the largest study, Jolivet et al., which contained 63% of the total meta-analysis population. Studies which screened for MDRO more than weekly had higher incidence rates for gut acquisition of MDRO compared to those that screened less than weekly (16.8 (95% CI: 12.6–22.6) vs. 10.2 (95% CI: 5.3–19.4) per 1000 patient-days respectively). The incidence rate for acquiring MDRO was also substantially higher for NP-GN than PA (11.8 (95% CI: 7.6–18.2) vs. 4.6 (95% CI: 1.3–16.6) per 1000 patient-days respectively).

**Table 3:** Pooled outcomes, stratified by organism and other study factors

	Studies (N)	Population (N)	Admission colonization (N, %)	Acquired colonization (N, %)	Incidence rate per 1000 patient-days (95% CI)*
Overall outcomes	14	37,305	3,007 (8%)	1669 (5%)	12.2 (8.1–18.6)
Excluding Jolivet et al	13	13,882	1,340 (10%)	1009 (8%)	12.6 (8.0–19.8)
Organism					
NP-GN	13	36,891	2,914 (8%)	1557 (5%)	11.8 (7.6–18.2)
PA	3	1113	41 (4%)	81 (8%)	4.6 (1.3–16.6)
VRE	1	481	63 (13%)	31 (7%)	4.6 (3.2–6.4)
Screening approach					
Weekly	9	35,236	2,746 (8%)	1478 (5%)	10.2 (5.3–19.4)
More than weekly	5	2069	261 (13%)	191 (11%)	16.8 (12.6–22.6)
Continent					
Europe	10	28,103	2,129 (8%)	1326 (5%)	13.4 (7.9–22.7)
Outside Europe	4	9202	878 (10%)	343 (4%)	8.8 (6.8–11.4)
Data collection period					
Majority pre-2013	10	36,188	2,875 (8%)	1454 (4%)	11.6 (7.1–19.1)
Majority post-2013	4	1,117	132 (12%)	139 (14%)	14.5 (5.9–35.5)
Organism identification method					
MALDI	5	24,596	1,830 (7%)	889 (4%)	17.2 (9.9–29.8)
Other	9	12,709	1177 (9%)	780 (7%)	10.5 (6.1–18.0)

NP-GN = non-*Pseudomonas* gram-negative organism. PA = *Pseudomonas aeruginosa*. VRE = vancomycin-resistant *Enterococcus*

*Incidence rate was calculated without Qin et al. [39] given that incidence rate could not be calculated for that study, while all other columns do include data from Qin et al. [39] Admission colonization is a prevalence out of the total population while acquired colonization is an incidence out of the population at risk

### Studies Reporting multiple specific timepoints

**Fig. 3:**
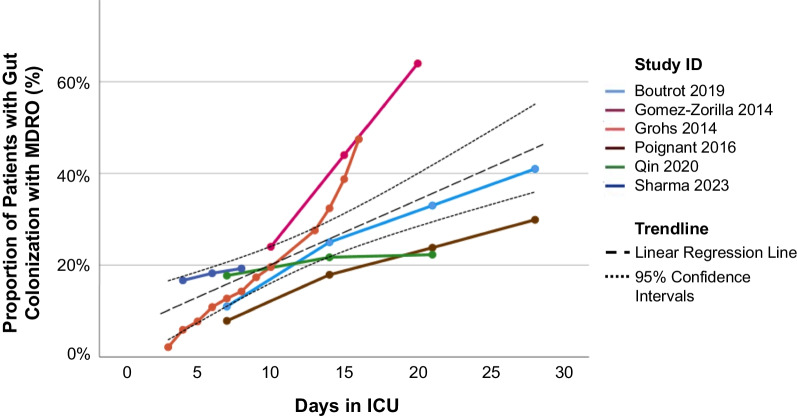
Cumulative proportion of multidrug resistant organisms (MDRO) bacteria over time in the ICU, among the six studies which reported the prevalence of gut acquisition of MDRO at multiple timepoints. Each study has been connected with colored lines and a linear regression line with 95% confidence intervals (dotted black lines) has been added which incorporates all of the data

There were six studies which reported the prevalence of gut MDRO colonization at multiple predetermined timepoints (e.g., prevalence of colonization at ICU Day 7, prevalence of colonization at ICU Day 14, etc.) (Fig. 3). Among these six studies, there was evidence of a steadily increasing proportion of patients with MDRO gut colonization through up to 30 days of ICU hospitalization (linear trend with an increase of 1.41% per day, R^2^ = 0.50, *p* < 0.01). Only Grohs et al. [23] accounted for the possibility of loss of colonization, while the 5 remaining studies assumed continuous colonization after a positive screening result.

### Quality and risk of *Bias*

Studies were assessed for quality and clarity using the 14-point Quality Assessment Tool of the National Institutes of Health [22]. Overall, quality was rated as good to fair for the majority of studies (Supplemental Figures S8 & S9). Specific areas with poor quality across studies included lack of adequate sample size justification and lack of blinding of outcome, with colonization results generally accessible to the treating clinical team. Only Grohs et al. [23] obtained additional rectal swabs to confirm gut MDRO colonization among patients that tested positive for colonization. Last, while the Egger’s test showed insignificant results for the funnel plot asymmetry (p = 0.805), the funnel plot of the standard error against the log incidence rate suggested possible publication bias against small studies which reported high incidence rates (Supplemental Figure S10).

## Discussion

This study reported on the incidence rate of MDRO gut colonization among patients in the ICU who were not colonized with an MDRO at admission. The prevalence of gut colonization at ICU admission and/or discharge has been studied in the past, but exactly *when* colonization occurs during ICU hospitalization has received less attention. Unlike previous meta-analyses, we were able to report on gut acquisition of MDRO in multiple ways: as a proportion of patients newly colonized during ICU hospitalization, as an incidence rate in patient-days, and in terms of median time to acquisition. Overall, we found 12.2 patients per 1000 patient-days acquired gut colonization with MDRO during ICU hospitalization and the risk of becoming colonized increased linearly with increased time in the ICU. Across studies, the median time to acquisition of colonization after ICU admission ranged from 4 to 26 days. Interestingly, there was less flattening of the curve for acquisition than one might expect, with a quasi-linear relationship between days spent in the ICU and prevalence of colonization. The risk of MDRO gut colonization varied by pathogen, with substantially higher rates observed among non-*Pseudomonas* Gram negatives compared to *Pseudomonas* spp. Pathogen colonization rates were also higher when screening was done at least weekly compared to less frequently, and when MALDI was used for pathogen identification compared to alternatives methods. In addition to the observed risk of MDRO gut colonization over the course of their ICU stay, 8% of patients were already gut colonized with MDRO at the time of ICU admission. The findings from this meta-analysis have important implications for the design of future clinical trials that evaluate measures directed at reducing gut colonization and subsequent infections by MDROs. Combined, the results suggest that interventions aiming to prevent or ameliorate MDRO gut colonization may be best deployed early during ICU admission and may need to be continued throughout ICU hospitalization. Our findings also highlight the heterogeneity of prior studies and the need for further research in this area.

Our findings align with prior meta-analyses [9, 12, 24]. Detsis et al. [9] found that the pooled incidence of ICU-acquired extended-spectrum beta-lactamase (ESBL)-producing Enterobacteriaceae colonization was 7%. Another meta-analysis by D’Agata et al. [8] found that the incidence of ICU-acquired colonization ranged from 4 to 29%. This wide range across studies—which was seen in our meta-analysis as well—may be due to baseline differences in populations or to approaches to measuring colonization. Another meta-analysis, focused on VRE, found that approximately 9% of patients were colonized on ICU admission to the ICU and another 9% acquired colonization during ICU hospitalization, similar to our results [10]. Our study found that the prevalence of colonization on ICU admission was higher than colonization acquired in the ICU for NP-GN but not for *Pseudomonas*. Similarly, Arzilli et al. [12] found that the prevalence of MDR Gram-negative gut colonization on hospital admission was 14% and that 9% acquired colonization, with variability based on organism. In our study and in other studies, the prevalence of MDRO gut colonization is high at ICU admission, with another substantial fraction of patients acquiring MDRO while hospitalized.

In sub-group analyses, we found that the incidence rate of gut colonization was higher among studies conducted within the past ten years, compared to those conducted from ten to 20 years ago. One of our largest included studies, Jolivet et al., reported on 18 years of data and found an increasing prevalence in ESBL-producing organisms from 1997 to 2015 [25]. Arzilli et al. observed that gut colonization prevalence was lowest before 2010, highest during the years 2010–2014, and decreased after 2014 [12]. Detsis et al. also found evidence of increasing MDRO gut colonization over time [9]. This data confirms the prioritization of WHO and other monitoring organizations. Our study also found that more frequent screening was associated with an increased detection of new colonization. Culture-based screening may have high false negative rates [26], and it is possible that studies under-represented the true rates of gut acquisition of MDRO. False positives are possible as well, and only one study in this meta-analysis used a tandem swabbing approach to confirm colonization.

The timing of gut colonization during ICU stay has not been studied as extensively as the prevalence, so it is less obvious how our results fit with previous studies. Detsis et al. found a pooled mean time from ICU admission to colonization of 11 days among three studies [9]; D’Agata et al. similarly found the median time to acquisition of a gut MDRO in the ICU ranged from 6 to 11.5 days [8]. These findings are similar to our result of a median time to acquisition ranging from 4 to 26 days. Future trials in this area may opt for study designs which deliver their interventions early during the ICU stay, which would fit into a broader ICU paradigm of early intervention (e.g. early antibiotic administration for sepsis) [27, 28].

This study has several limitations which should be considered when interpreting the findings. There was significant heterogeneity between included studies which raises questions about the validity of pooling results. We have produced pooled results because we believe that these results will help to better focus future studies and will highlight gaps in knowledge. These pooled results must be interpreted cautiously and they highlight the need for further standardization of reporting in future research. We were also unable to report on patient factors which may influence acquisition of gut MDRO such as prior or cumulative antibiotic exposure, immunodeficiency, or local prevalence of MDRO [9, 29, 30]. Additional research is needed on the timing of colonization acquisition based on these important risk factors in order to develop more targeted interventions. We did not separately analyze results based on perianal vs rectal swabs. While there is evidence to suggest results of these tests are similar [31], it is possible that perianal swabs include skin flora that rectal swabs do not which could affect the results of this study. Because the included studies reported a variety of measures (incidence, prevalence, mean, median, etc.), several assumptions had to be made during data extraction and synthesis in order to pool data together. We believe that these assumptions are reasonable, and that they do not detract from the validity of results. Most of the studies were from Europe or the U.S., and generalizability to other regions is unclear. Finally, assessment of colonization may be affected by a high false negative rate [26] and intermittent colonization as not all patients who are colonized will consistently remain colonized throughout their ICU stay [23].

## Conclusion

Acquisition of gut MDRO was common in the ICU and there is a positive linear association between the proportion of patients with gut MDRO colonization and time spent in ICU through 30 days of ICU hospitalization. These data may guide future interventions seeking to prevent or ameliorate colonization. Specifically, such interventions will likely need to intervene early during the ICU stay and be maintained continuously to be effective.

## Availability of data and raw materials

All data generated or analyzed during this study are included in this published article [and its supplementary information files].

### Supplementary Information

**Additional file 1**.
