# Supplementary material for: Gut colonization with multidrug resistant organisms in the intensive care unit: a systematic review and meta-analysis
Source: Crit Care. 2024 Jun 28;28:211. doi: 10.1186/s13054-024-04999-9 (PMC11214232; doi:10.1186/s13054-024-04999-9)
Supplement: Supplementary file 1 — Additional file 1. [file 13054_2024_4999_MOESM1_ESM.docx]

**SUPPLEMENTAL TABLES**

**Supplemental Table 1**. Search strategy for each database

**Search strategy for Pubmed on November 8, 2023**

| Search Framework | Search terms | Number of results |
| --- | --- | --- |
| Condition: colonization | "Bacterial Adhesion"[Mesh] OR "Carrier State"[Mesh] OR coloniz*[tiab] OR colonis*[tiab] OR carriage*[tiab] OR carrier*[tiab] OR carry*[tiab] OR decoloni*[tiab] | 656,609 |
| Condition: drug resistant organisms | “Drug Resistance, Bacterial"[Mesh] OR "multidrug resist*"[tiab] OR "multi drug resist*"[tiab] OR "multiple drug resist*"[tiab] OR mdr[tiab] OR mdrs[tiab] OR "multiresistant bacteri*"[tiab] OR "antimicrobial resist*"[tiab] OR ("antimicrobial" AND "resist*"[tiab]) OR "antibacterial drug resist*"[tiab] OR (“antibacterial” AND “resist”[tiab]) OR "antibiotic resistant bacteri*"[tiab] OR (“antibiotic” AND “resist*”[tiab]) OR "resistant multidrug*"[tiab] OR  esbl*[tiab] OR "extended spectrum beta lactam*"[tiab] OR "beta lactamase*"[tiab] OR "beta-Lactam Resistance"[Mesh] OR "beta-Lactamases"[Mesh] OR "beta-Lactams"[Mesh] OR  "carbapenemase"[tiab] OR carbapenem*[tiab] OR carbenicillinase*[tiab] OR carbenicillinase*[tiab] OR  "extended spectrum cephalosporin*"[tiab] OR "expanded spectrum cephalosporin*"[tiab] OR "cephalexin amidase*"[tiab] OR cephalosporinas*[tiab] OR “ceftriaxone resist*”[tiab] OR  “Vancomycin Resistance"[Mesh] OR "vre"[tiab] OR “vancomycin-resist*”[tiab] OR “vancomycin resist*”[tiab] OR  enterobacter*[tiab] OR acinetobacter*[tiab] OR "A. baumannii"[tiab] OR pseudomon*[tiab] OR "gram negative"[tiab] OR "gram-negative"[tiab] OR enterococc*[tiab] OR “Enterococcus faecium”[tiab] OR “E. faecium”[tiab] OR “E. coli”[tiab] OR “Klebsiella”[tiab] | 724,757 |
| Context: Gut | "Gastrointestinal Tract"[Mesh] OR “Gastrointestinal Microbiome"[Mesh] OR "Feces"[Mesh] OR stool[tiab] OR feces[tiab] OR faeces[tiab] OR fecal*[tiab] OR faecal*[tiab] OR intestin*[tiab] OR gastrointestin*[tiab] OR digestiv*[tiab] OR gut[tiab] OR guts[tiab] OR anal[tiab] OR anus[tiab] OR rectum[tiab] OR rectal*[tiab] OR perineum[tiab] OR perineal*[tiab] OR perianal*[tiab] | 1,538,292 |
| Population | “Intensive Care Units”[Mesh] OR “ICU”[tiab] OR “Intensive Care Unit”[tiab] AND (alladult[Filter] OR adult[Filter] OR middleagedaged[Filter] OR middleaged[Filter] OR aged[Filter] OR 80andover[Filter] OR youngadult[Filter]) | 84,842 |
| Study design | "Cohort Studies"[Mesh] OR cohort[tiab] OR "natural history"[tiab] OR "follow up" OR “follow-up” | 4,665,620 |
| Combined terms | All the above terms connected with AND & limited to 2010 - 2024 | 117 |

**Search strategy for Embase on November 8, 2023**

| Search Framework | Search terms | Number of results |
| --- | --- | --- |
| Condition: colonization | 'bacterium adherence'/exp OR 'disease carrier'/exp 'colonization'/exp OR coloniz*:ti,ab,kw OR colonis*:ti,ab,kw OR carriage*:ti,ab,kw OR carrier*:ti,ab,kw OR carry*:ti,ab,kw OR decoloni*:ti,ab,kw | 780,897 |
| Condition: drug resistant organism | 'drug resistance'/exp OR 'drug resistance' OR (('drug'/exp OR drug) AND ('resistance'/exp OR resistance)) OR 'multidrug resist*':ti,ab,kw OR 'multi drug resist*':ti,ab,kw OR mdr:ti,ab,kw OR mdrs:ti,ab,kw OR 'multiresistant bacteri*':ti,ab,kw OR 'antimicrobial resist*':ti,ab,kw OR 'antimicrobial resistance'/exp OR (('antimicrobial'/exp OR antimicrobial) AND ('resistance'/exp OR resist*)) OR 'antibacterial drug resist*':ti,ab,kw OR 'antibacterial resistance'/exp OR (('antibacterial'/exp OR antibacterial) AND ('resistance'/exp OR resist*)) OR 'antibiotic resistant bacteri*':ti,ab,kw OR ('antibiotic' AND 'resist*':ti,ab,kw) OR 'resistant multidrug*':ti,ab,kw OR  esbl*:ti,ab,kw OR 'extended spectrum beta lactam*':ti,ab,kw OR 'beta lactamase*':ti,ab,kw OR 'beta-lactam resistance'/exp OR 'beta-lactamases'/exp OR 'beta-lactams'/exp OR  'carbapenemase':ti,ab,kw OR carbapenem*:ti,ab,kw OR carbenicillinase*:ti,ab,kw OR carbenicillinase*:ti,ab,kw OR  'extended spectrum cephalosporin*':ti,ab,kw OR 'expanded spectrum cephalosporin*':ti,ab,kw OR 'cephalexin amidase*':ti,ab,kw OR cephalosporinas*:ti,ab,kw OR 'ceftriaxone resist*':ti,ab,kw OR  'vancomycin resistance'/exp OR 'vre':ti,ab,kw OR 'vancomycin-resist*':ti,ab,kw OR 'vancomycin resist*':ti,ab,kw OR  enterobacter*:ti,ab,kw OR acinetobacter*:ti,ab,kw OR 'a. baumannii':ti,ab,kw OR pseudomon*:ti,ab,kw OR 'gram negative':ti,ab,kw OR ‘gram-negative’:ti,ab,kw OR enterococc*:ti,ab,kw OR 'enterococcus faecium':ti,ab,kw OR 'e. faecium':ti,ab,kw OR ‘E. coli’:ti,ab,kw OR ‘Klebsiell’:ti,ab,kw | 1,562,591 |
| Context: gut | 'gastrointestinal tract'/exp OR 'gastrointestinal microbiome'/exp OR 'feces'/exp OR stool:ti,ab,kw OR feces:ti,ab,kw OR faeces:ti,ab,kw OR fecal*:ti,ab,kw OR faecal*:ti,ab,kw OR intestin*:ti,ab,kw OR gastrointestin*:ti,ab,kw OR digestiv*:ti,ab,kw OR gut:ti,ab,kw OR guts:ti,ab,kw OR anal:ti,ab,kw OR anus:ti,ab,kw OR rectum:ti,ab,kw OR rectal*:ti,ab,kw OR perineum:ti,ab,kw OR perineal*:ti,ab,kw OR perianal*:ti,ab,kw | 1,538,129 |
| Population | ('intensive care units'/exp OR 'icu':ti,ab,kw OR 'intensive care unit':ti,ab,kw) AND ([adult]/lim OR [aged]/lim OR [middle aged]/lim OR [very elderly]/lim OR [young adult]/lim) | 212,986 |
| Study design | 'cohort studies'/exp OR cohort:ti,ab,kw OR 'natural history':ti,ab,kw OR 'follow up' OR ‘follow-up’ | 5,665,277 |
| Combined terms | All the above terms connected with AND & limited to 2010 - 2024 | 210 |

**Search strategy for Web of Science on November 8, 2023**

| Search Framework | Search terms | Number of results |
| --- | --- | --- |
| Condition: colonization | TS = ("colonis*" OR "coloniz*" OR "colony" OR "colonies" OR "carriage*" OR "carrier*" OR "carry*" OR “decoloni*” OR “bacterial adhesion”) | 1,617,957 |
| Condition: drug resistant organisms | TS = ("multidrug resist*" OR "multi drug resist*" OR "multiple drug resist*" OR mdr OR mars OR "multiresistant bacteri*" OR "antimicrobial resist*” OR ("antimicrobial" AND "resist*") OR "antibacterial drug resist*" OR (“antibacterial” AND “resist”) OR "antibiotic resistant bacteri*" OR (“antibiotic” AND “resist*”) OR "resistant multidrug*" OR  esbl* OR "extended spectrum beta lactam*" OR "beta lactamase*" OR "beta-Lactam Resistance" OR "beta-Lactamases" OR "beta-Lactams" OR  "carbapenemase" OR carbapenem* OR carbenicillinase* OR carbenicillinase* OR  "extended spectrum cephalosporin*" OR "expanded spectrum cephalosporin*" OR "cephalexin amidase*" OR cephalosporinas* OR “ceftriaxone resist*” OR  “Vancomycin Resistance" OR "vre" OR “vancomycin-resist*” OR “vancomycin resist*” OR  enterobacter* OR acinetobacter* OR "A. baumannii" OR pseudomon* OR "gram negative" OR "gram-negative" OR enterococc* OR “Enterococcus faecium” OR “E. faecium” OR “E. coli” OR “Klebsiella”) | 805,146 |
| Context: Gut | TS = ("Gastrointestinal Tract" OR “Gastrointestinal Microbiome" OR "Feces" OR stool OR feces OR faeces OR fecal* OR faecal* OR "Intestin*" OR gastrointestin* OR digestiv* OR gut OR guts OR anal OR anus OR rectum OR rectal* OR perineum OR perineal* OR perianal*) | 1,248,896 |
| Population | TS = (“Intensive Care Units” OR “ICU” OR “Intensive Care Unit”) NOT TS = ('pediatric' OR 'paediatric' OR 'neonat*' OR ‘newborn’ OR ‘infant’ OR ‘child’ OR 'adolescent') | 151,749 |
| Study design | TS = ("Cohort Studies" OR cohort OR "natural history" OR "follow up" OR followup) | 3,792,302 |
| Combined terms | All the above terms connected with AND & limited to 2010 - 2024 | 155 |

**Supplemental Table 2**. Detailed descriptions of included studies

| **Study** | **Swab location** | **Swab frequency** | **Inclusion criteria** | **Organism identification method** | **Antibiotic susceptibility method** |
| --- | --- | --- | --- | --- | --- |
| Ajao 2013 | peri-anal | admission;  weekly;  discharge | - Medical or surgical ICU - No previous ESBL+ clinical cultures - ESBL negative swab at admission - ICU length of stay of at least 48 hours | MacConkey agar with ceftazidime incubated for 24-28 hours then the VITEK II system (bioMerieux). | Disk diffusion |
| Alves 2016 | rectal | admission;  2x/week;  discharge | - Medical ICU - Consent obtained | Agar with ceftazidime incubated for 24 hours and MALDI-TOF. | Disk diffusion |
| Boutrot 2019 | rectal | admission;  weekly | - Surgical ICU - First admission to the ICU - Able to collect admission sample and at least one surveillance sample | ChromID ESBL agar (bioMerieux) incubated for 12 hours and microflex MALDI-TOF (Bruker). | Disk diffusion |
| Gomez-Zorrilla 2014 | rectal | admission;  weekly | - Medical or surgical ICU - ICU stay of ≥ 48 hours | MicroScan system (Dade International). | Broth microdilution |
| Grohs 2014 | rectal | admission;  daily;  discharge | - Medical or surgical ICU - Consent obtained | ChromID ESBL agar (bioMerieux) incubated for 18 hours then microflex MALDI-TOF (Bruker). | Disk diffusion |
| Jolivet 2020 | rectal | admission;  weekly | - All ICUs | Drigalski agar (Bio-Rad) with cefotaxime or ChromID ESBL agar (bioMerieux) then API 20E strips (bioMerieux) or MALDI Biotyper (Bruker). | Disk diffusion |
| Marchenay 2015 | rectal;  throat swab/tracheal | admission;  weekly | - Medical or surgical ICU - ICU stay of ≥ 48 hours - No colonizing MDR-GN within 48 hours | Drigalski agar (Oxoid) with an ertapenem disc (Biorad). | Disk diffusion;  E-test |
| Papadimitriou-Olivgeris 2015 | rectal | admission; weekly | - All ICUs | Selective chromogenic agar then the VITEK II system (bioMerieux). | Disk diffusion |
| Poignant 2016 | rectal | admission; weekly | - Medical or surgical ICU - First admission to the ICU - ICU length of stay of ≥ 48 hours | Selective agar incubated for 12 hours. | Disk diffusion |
| Qin 2020 | rectal;  nasopharyngeal | admission; weekly | - Neurological ICU - Age ≥ 14 years - Expected to spend ≥ 3 days in ICU | Mueller-Hinton agar incubated for 12 hours then MALDI-TOF. | Broth microdilution |
| Razazi 2012 | rectal | admission; 2x/week | - Medical ICU - First admission to the ICU | Selective chromogenic agar. | Disk diffusion |
| Sharma 2023 | peri-rectal | admission; every other day until day 8 | - Medical or surgical ICU | MacConkey agar with ertapenem disk and immersed in trypticase soy broth with meropenem disk. | Disk diffusion |
| Thiébaut 2012 | rectal | admission;  2x/week | - Medical or surgical ICU - First admission to the ICU | Biplate containing MacConkey agar with ceftazidime and Drigalski agar with cefotaxime. | Disk diffusion;  E-test |
| Torres-Gonzalez 2015 | rectal | admission;  weekly | - All ICUs - Patients with: abdominal sepsis, transfer from another institution, solid organ or bone marrow transplant, CRE infection, or roommate with CRE infection | Trypticase soy broth with ertapenem disk. | Disk diffusion |

**SUPPLEMENTAL FIGURES**

**Supplemental Figure 1.** Proportion of intensive care unit (ICU) patients with gut colonization with multidrug resistant organisms (MDRO) at ICU admission and at the median time to acquisition for patients who were negative for MDRO at admission but subsequently acquired them.

**
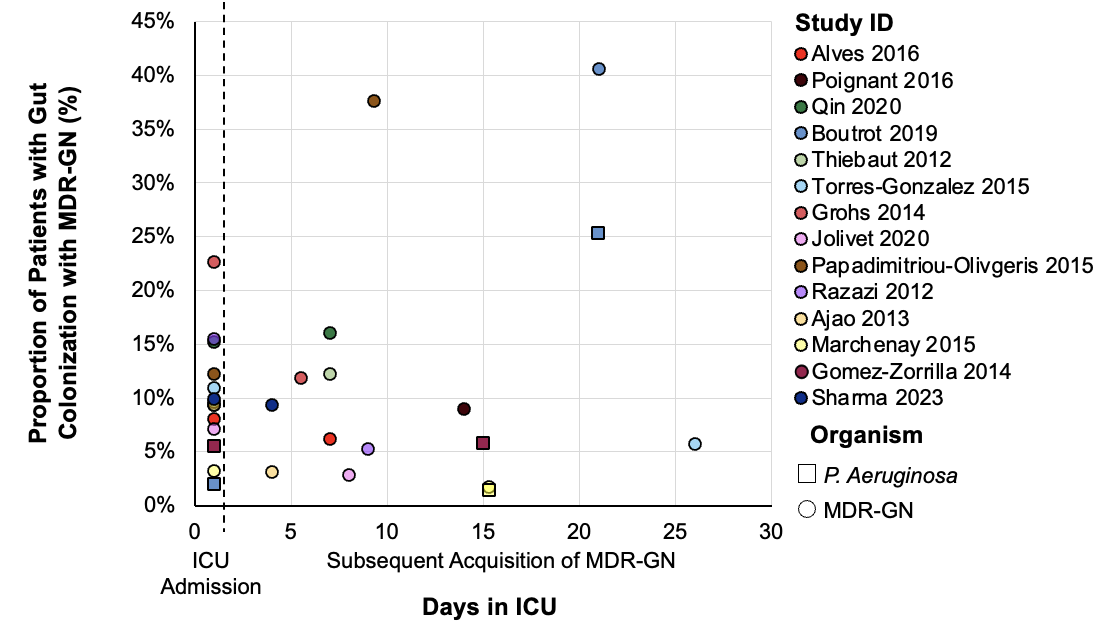
**

**Supplemental Figure 2**. Forest plot of incidence rate of gut acquisition of MDRO per 1,000 patient-days stratified by size of study.**
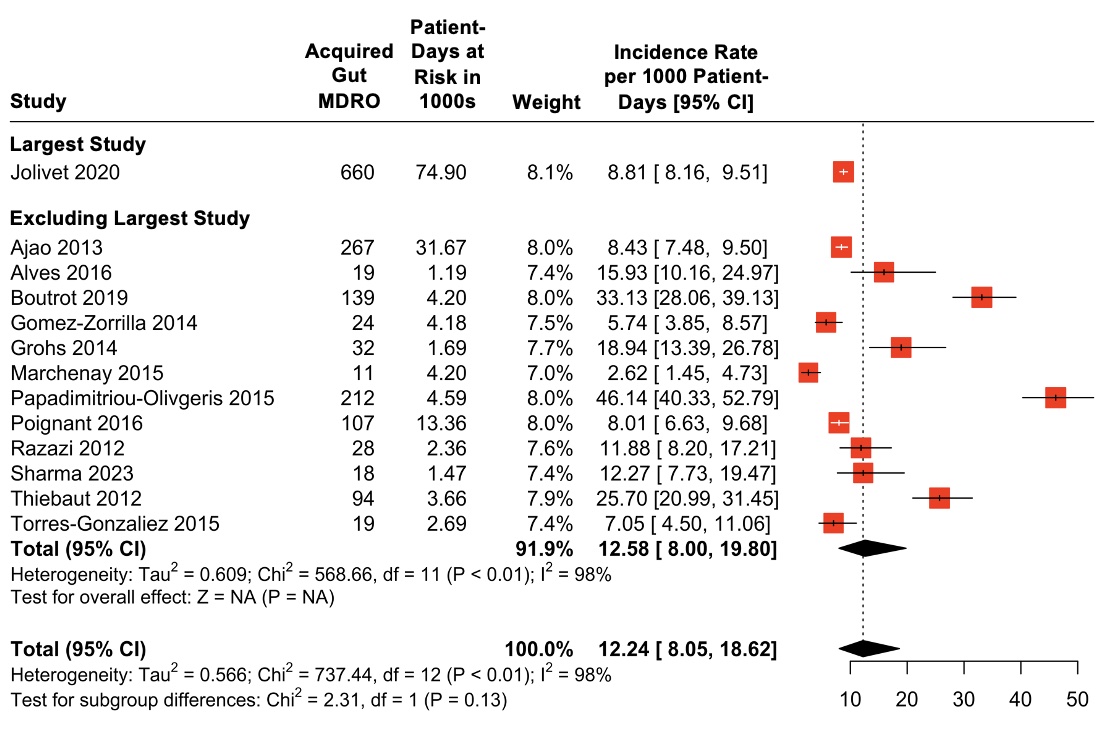
**

**Supplemental Figure 3**. Forest plot of incidence rate of gut acquisition of MDRO per 1,000 patient-days stratified by colonizing organism.

**
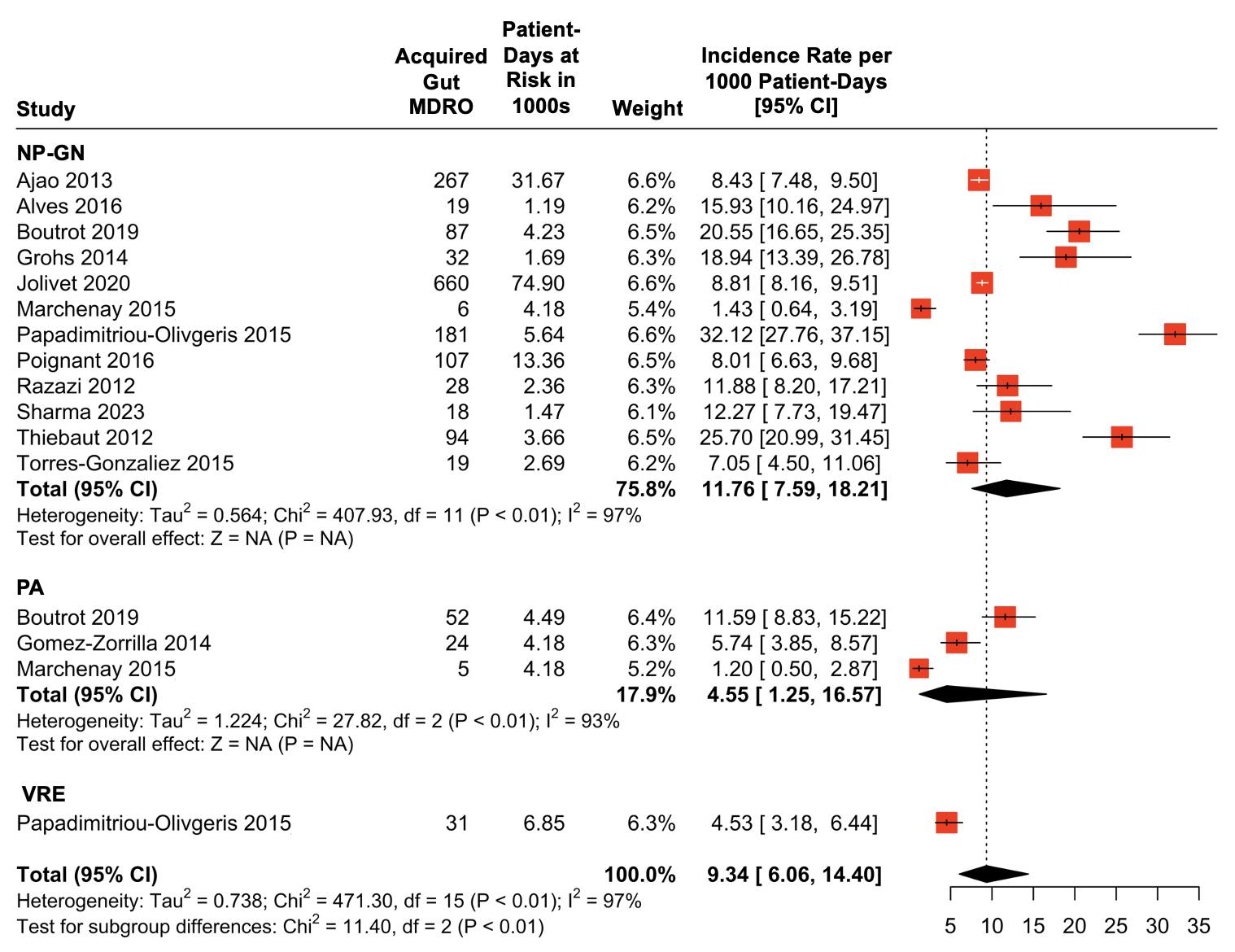
**

**Note**: NP-GN = non-*Pseudomonas* gram-negative organism. PA = *Pseudomonas aeruginosa*. VRE = vancomycin-resistant *Enterococcus*.

**Supplemental Figure 4**. Forest plot of incidence rate of gut acquisition of MDRO per 1,000 patient-days stratified by frequency of screening. Screening weekly indicates screening for colonization was performed once per week and screening more than weekly indicates screening for colonization was performed more frequently than once per week (e.g. twice per week or every day).

**
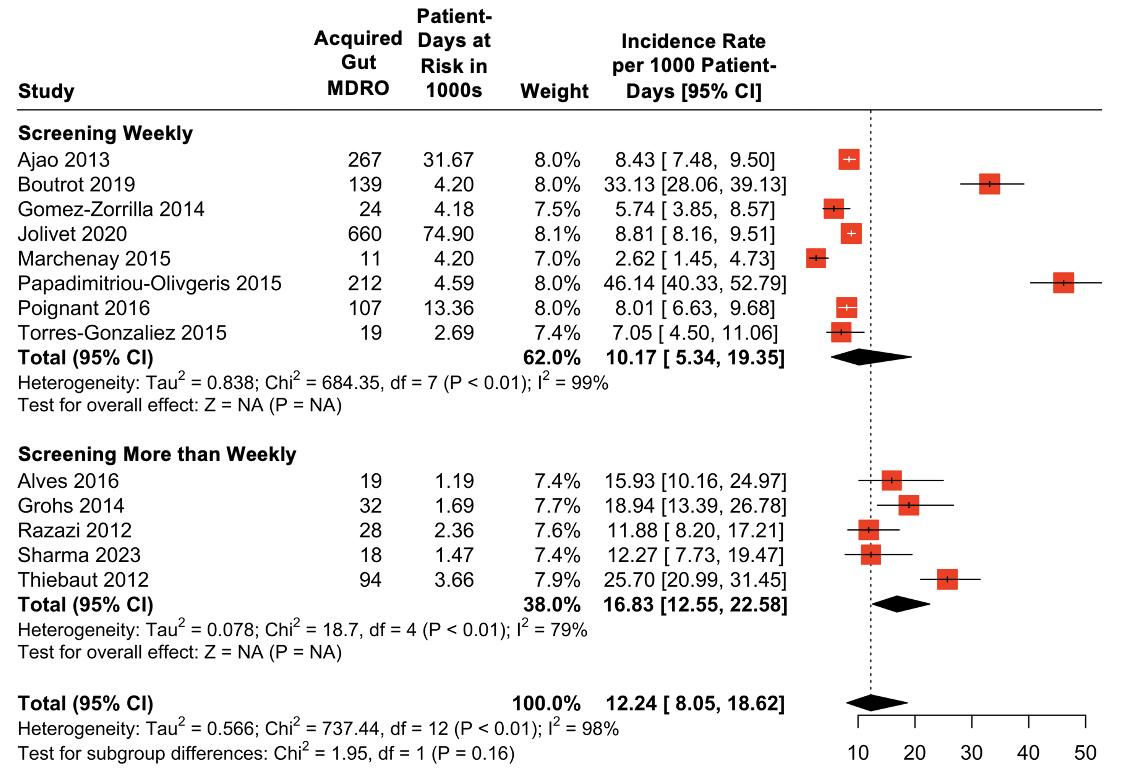
**

**Supplemental Figure 5**. Forest plot of incidence rate of gut acquisition of MDRO per 1,000 patient-days stratified by continent.

**
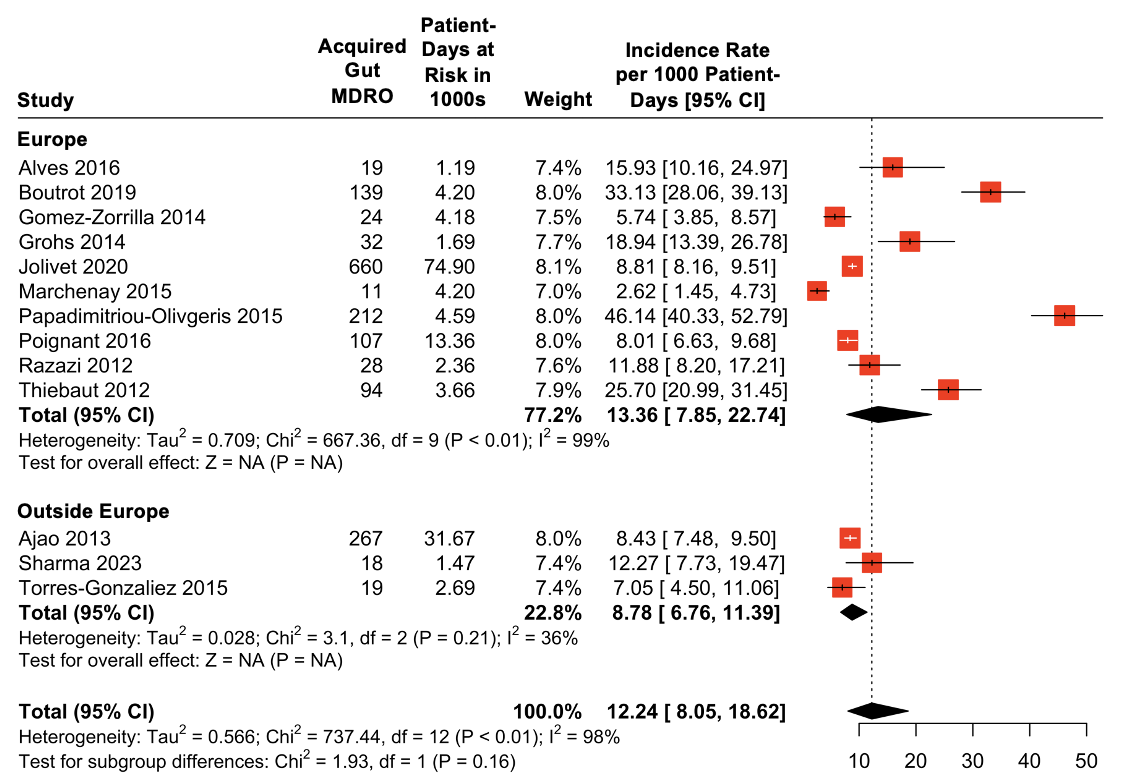
**

**Supplemental Figure 6**. Forest plot of incidence rate of gut acquisition of MDRO per 1,000 patient-days stratified by year of data collection. Majority pre-2013 indicates more than 50% of the data was collected in the year 2013 or earlier. Majority post-2013 indicates more than 50% of the data was collected in the year 2014 or later.

**
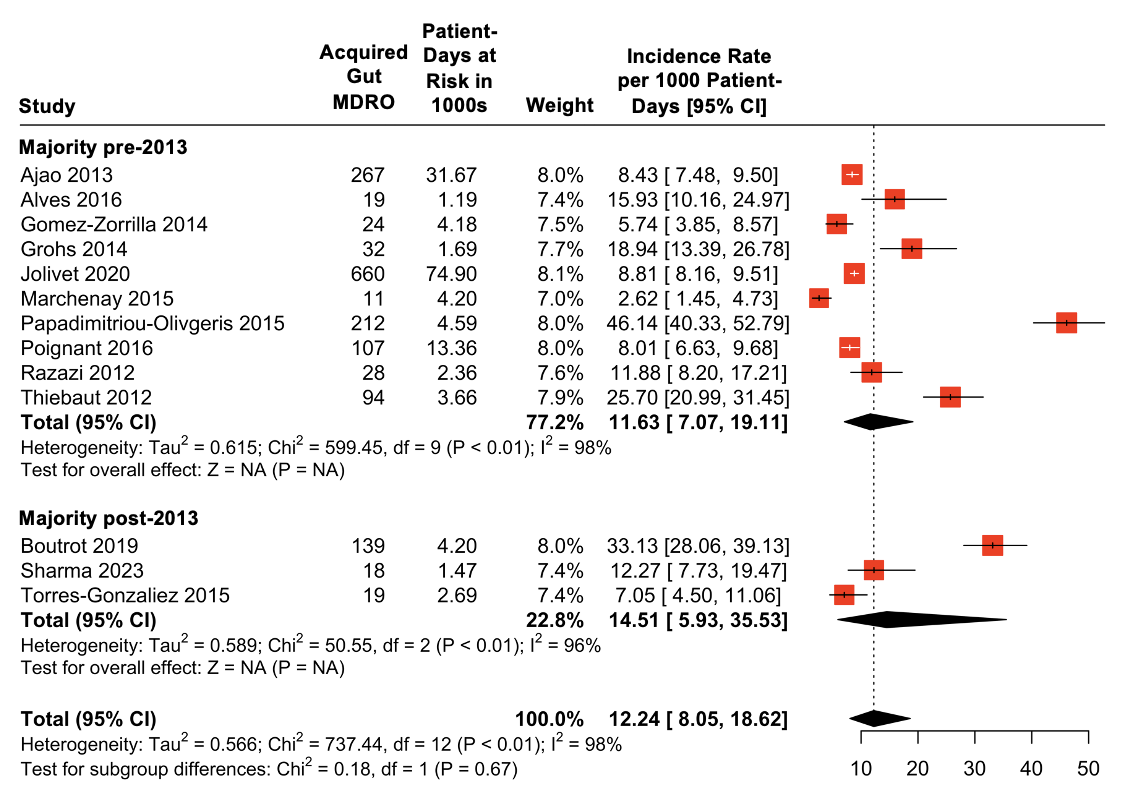
**

**Supplemental Figure 7**. Forest plot of incidence rate of gut acquisition of MDRO per 1,000 patient-days stratified by organism identification method.

**
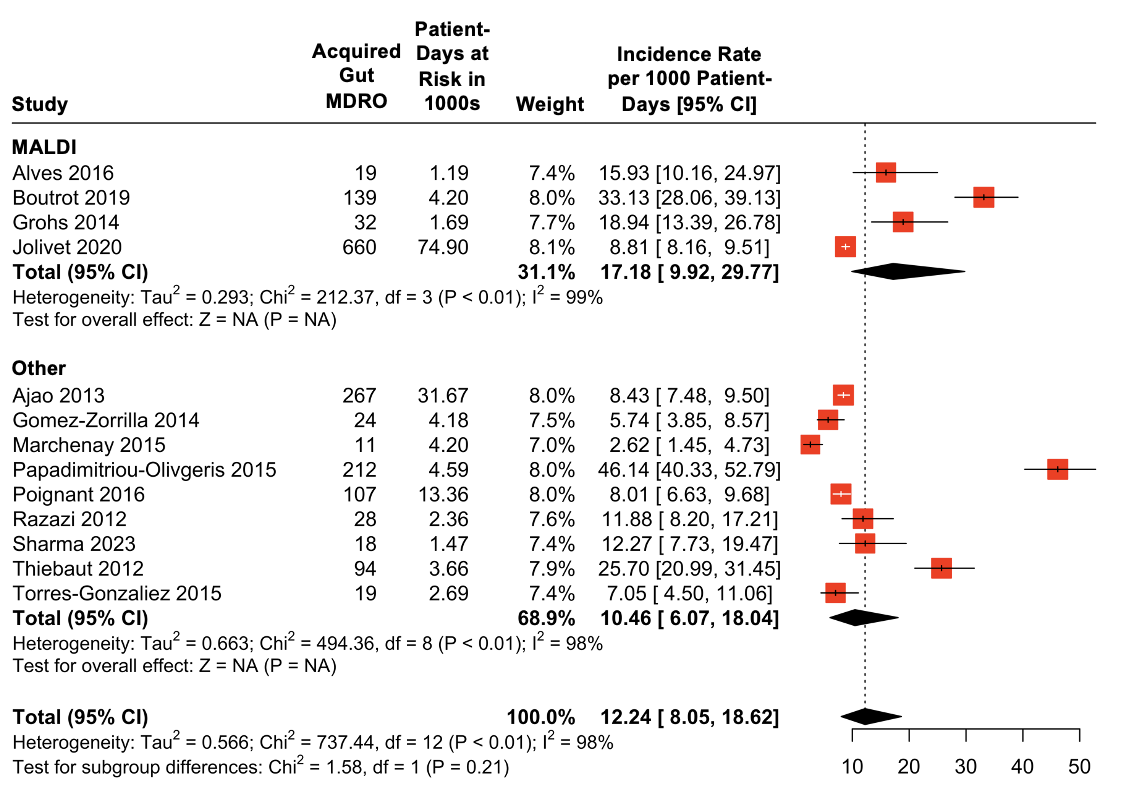
**

**Note:** MALDI = Matrix assisted laser desorption/ionization

**Supplemental Figure 8**. Quality Assessment traffic light plot

**Note**: The green “+” indicates good quality; the yellow “-“ indicates fair quality; the red “x” indicates poor quality; the grey “?” indicates the quality is unclear. See **Supplemental Methods** for further details.

**Supplemental Figure 9**. Quality assessment cumulative results

**Study Quality**

**Note**: See **Supplemental Methods** for additional information on each question.

**Supplemental Figure 10**. Funnel plot of incidence rate against standard error for publication bias (Egger’s test p=0.805)


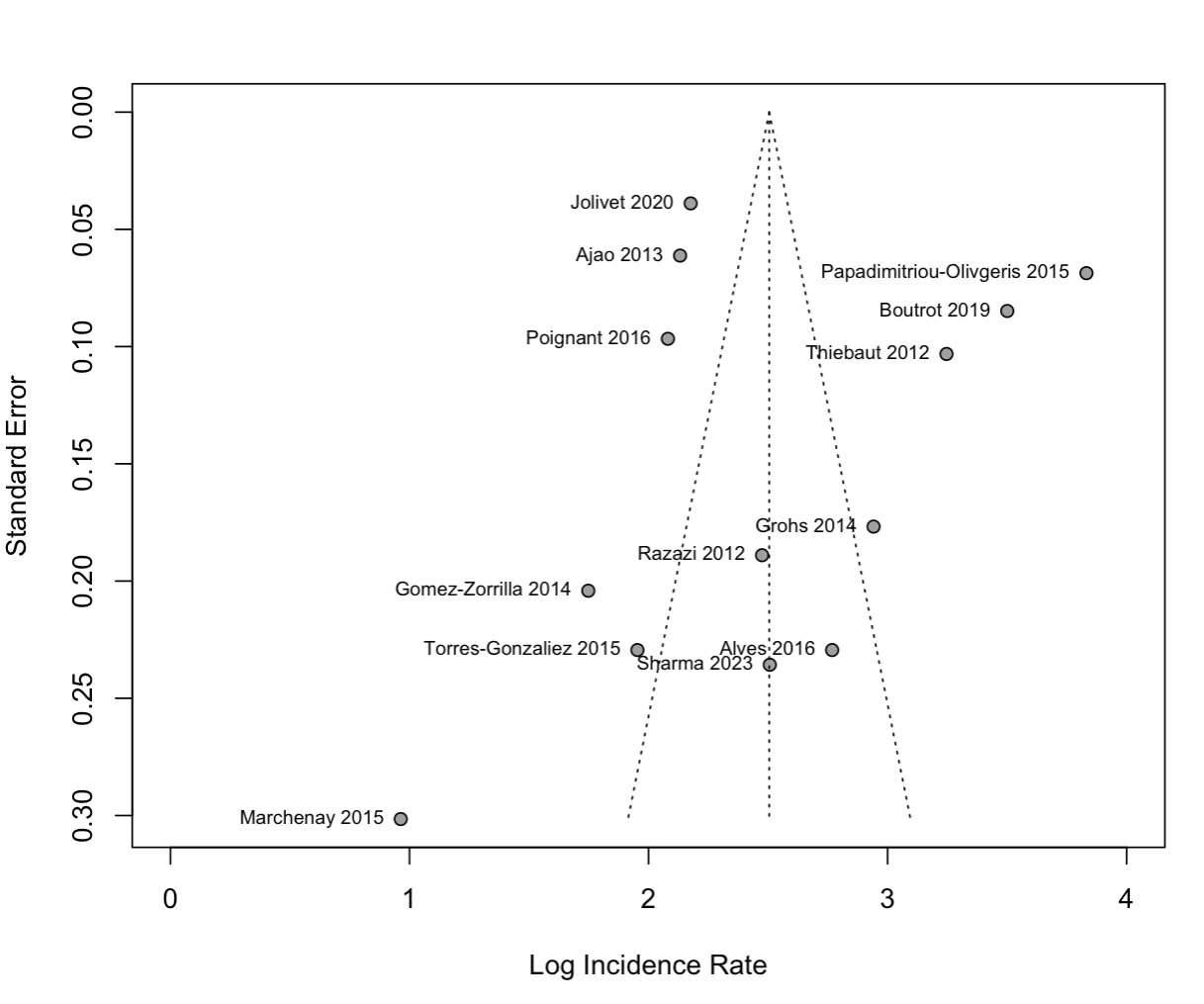


**Supplemental Methods**

***Study Inclusion Criteria***

Studies were required to report on MDRO gut colonization based on stool samples or rectal swabs that were gathered at ICU admission (within 48 hours) and at one or more subsequent timepoint. Studies that did not report the prevalence of gut colonization on admission to the ICU, or which did not report a measure of the time between admission to gut acquisition of MDRO were excluded. To enhance generalizability, studies were further required to perform sampling on consecutive ICU patients (i.e., they were excluded if they focused on a specific subset of ICU patients, such as cirrhotic or immunocompromised patients), and to report on at least 50 participants at risk for gut MDRO colonization. Because we sought to report on the “natural history” of colonization in the ICU, all studies with an intervention were excluded. Abstracts or studies lacking a published version of the full text in English were also excluded.

***Inclusion of Specific Studies***

Additional comments regarding inclusion of specific studies:

- Torres-Gonzalez *et al*.^42^ included a relatively small proportion of non-ICU patients in addition to ICU patients, often patients with abdominal sepsis and transplant patients. Most of these patients would meet criteria for an ICU depending on the institution, and we felt the study met our inclusion criteria.
- Qin *et al*.^38^ included a small proportion of patients age 14-18. While our review focuses on adult populations, given that the average patient age was in the 50s, comparable to other studies we felt this was still an adult ICU study rather than a pediatric ICU and that the study should be included in this meta-analysis.

***Data Extraction for Specific Studies***

Additional comments regarding data extraction for specific studies:

- Boutrot et al.^33^ Organisms were separated into 3 groups: ESBL-producing Enterobacteriaceae, AmpC hyperproducing Enterobacteriaceae, and *Pseudomonas aeruginosa*. For the purpose of our study, ESBL-producing Enterobacteriaceae and AmpC hyperproducing Enterobacteriaceae were grouped together into our non-*Pseudomonas* gram negative organism category. The median and interquartile range for time to colonization for ESBL-producing Enterobacteriaceae and AmpC hyperproducing Enterobacteriaceae was 13 (7-41) and 14 (6-20). The weighted mean of the two medians was 14. Because there was insufficient data to combine the IQRs, the IQR for AmpC hyperproducing Enterobacteriaceae was used. Additionally, Figure 1 from this paper was used to estimate the number of participants colonized at each timepoint. The proportion of patients colonized with at least one multi-drug resistant organism was estimated by measuring the height of the corresponding bar on the graph relative to the y-axis.
- Gomez-Zorilla *et al*.^34^ Table 2 was used to estimate median time to colonization which was not reported in the text of the Results section. While this table only accounts for a subset of all the patients that acquired colonization, it was assumed that time to colonization would be similar across the remaining patients. Figure 2 was used to estimate the proportion of patients colonized at 3 timepoints: 10 days, 15 days, and 20 days. Gomez-Zorilla *et al*. separated their organisms of interest into 3 groups: non-drug resistant *Pseudomonas*, multi-drug resistant *Pseudomonas* (excluding XDR cases), and XDR *Pseudomonas* (defined as susceptible to 2 or less drugs and resistant to all others). For our meta-analysis, multi-drug resistant *Pseudomonas* and XDR could both be included. However, because patients could be colonized with MDR and XDR simultaneously and there was likely considerable overlap based on similar rates of colonization within Figure 2, only the XDR proportion was included in our study.
- Grohs *et al*.^23^ Figure 2 was used to determine the number of patients that acquired colonization on each day. Due to our definition of colonization on admission as within the first 48 hours since admission, newly acquired cases were recorded starting on day 3 from ICU admission. Grohs *et al*. recorded colonization with ESBL-producing and high-level expressed AmpC cephalosporinase (HL-CASE)-producing Enterobacteriaceae separately. These groups were combined into the non-*Pseudomonas* gram negative group for our meta-analysis; therefore, the first colonization with either organism was recorded as the day of acquiring colonization. This data was used to calculate the median and IQR for time to acquisition. Because every other study we included did not reassess colonization after the first positive result, we assumed that a positive result indicated that patient was colonized for the remainder of their time in the ICU, although Figure 2 in this study suggests that some patients did test positive and then subsequently negative for gut colonization. This data also was used to determine the proportion of cases colonized at each time point. Because the rate of attrition from the ICU was not specified, it was estimated to be 1.5 patients per day based on the data in Figure 2. The acquired cases made up approximately 11% of the total study population, so it was assumed that 12.6 patients per day left the ICU (died or were discharged) from the total initial population. This was used to estimate the proportion of patients in the ICU colonized at each timepoint.
- Poignant *et al*.^37^ Figure 1 was used to determine the cumulative proportion of patients colonized at each timepoint as labeled on the x-axis.
- Qin *et al*.^38^ Figure 1 was used to estimate the number of participants colonized at each timepoint. Qin *et al*. separated participants that acquired carbapenem resistant *Klebsiella pneumoniae* into two groups: those that did not have any gut colonization on admission and those that were found to have carbapenem-susceptible *Klebsiella pneumoniae* on admission. For the purposes of our study, these groups were combined. The proportion of participants colonized at each timepoint was determined based on the numbers reported in Figure 1. Each follow-up timepoint was assumed to be one week based on the methods which describe “swabbing weekly” and on the relevant figure.
- Sharma *et al*.^40^ The following information was stated in the study: 13 CRE isolates were recovered on the 4^th^ day, 3 on the 6th day and 2 on the 8th day of ICU admission. Nineteen patients were colonized on ICU admission. Because the median ICU stay was greater than all the timepoints of data collection, it was assumed that there was no ICU attrition, so the total number of people in the ICU at each timepoint was assumed to be 192. Therefore, the cumulative proportion of people colonized at each time point was assumed to be the cumulative sum of all people colonized divided by the total number of people in the ICU.

***Calculations and Approximations***

***Means and Medians***

For studies that reported the mean but not the median time to acquisition, we assumed the distribution of data was normal and used the mean to approximate the median.

***Incidence Rate***

Incidence rate was approximated based on the time to acquisition and ICU length of stay, and expressed per 1,000 person-days, similar to the methods used by Willems et al.^3^ In this calculation, the person-days at risk was = (N_ICU acquired cases_ * Median Days to Acquisition) + (N _never acquired colonization_ * ICU Length of Stay)*.* The ICU length of stay specific for patients that did not acquire colonization was used if available. Otherwise, the ICU length of stay for all (combined) patients was used. The incidence rate was then calculated as follows:

Incidence Rate = N_ICU acquired cases_ / Person-Days at Risk * 1,000

***Risk of Bias Criteria***

Risk of bias criteria was based on NIH criteria as follows:

Q1. **Research objective**. Good = clearly stated

Q2. **Study Population**. Good = clearly defined in methods and described in results

Q3. **Participation rate**. Good = greater than 50% of eligible people participated; Poor = less than 30% of eligible people participated; Unclear = did not specify how frequently or successfully standard of care swabs

Q4. **Recruitment population**. Good = subjects recruited from same or similar populations; Fair = subjects recruited from similar populations, but with variations in time period or ICU characteristics

Q5. **Sample size justification**. Good = clearly justified sample size; Fair = results adjusted for sample size; Poor = no sample size justification

Q6. **Exposure assessed prior to outcome**. Good = colonization was assessed within 48 hours of ICU admission

Q7. **Sufficient timeframe to see an effect**. Good = patients followed for greater than 14 days after ICU admission while in the ICU; Fair = patients were followed for 7 to 14 days; Poor = patients followed for less than 7 days

Q8. **Levels of exposure**. Good = swabs taken more than 1x/week; Fair = swabs taken every 7 days; Poor = swabs taken less than every 7 days

Q9. **Exposure measure validity**. Good = site and frequency of swab specified + results separated by site of swab; Fair = site and frequency of swab specified in methods, but results not separated by type of swab

Q10. **Repeated exposure assessment**. Good = swab repeated within 24 hours; Poor = swab not repeated

Q11. **Outcome measures**. Good = clear methods and criteria for identifying bacterial growth; Fair = vague or non-specific criteria

Q12. **Blinding of outcome assessors**. Good = clinicians were blind to results of swabs; Poor = clinicians not blinded to results of swabs or swabs collected as standard of care; Unclear = studies did state if swabs were collected only for the study or as standard of care

Q13. **Follow-up rate**. Good = compliance greater than or equal to 50%; Fair = compliance was less than 50% but greater than 25%; Unclear = compliance rates were not explicitly stated

Q14. **Statistical analysis**. Good = results are adjusted for potential confounders; Fair = reported of adequate statistics; Unclear = no statistics section within the methods
